# Supplementary material for: Molecular Clustering Analysis of Blood Biomarkers in World Trade Center Exposed Community Members with Persistent Lower Respiratory Symptoms
Source: Int J Environ Res Public Health. 2022 Jul 1;19(13):8102. doi: 10.3390/ijerph19138102 (PMC9266229; doi:10.3390/ijerph19138102)
Supplement: Supplementary file 1 [file ijerph-19-08102-s001.zip › Supplemental Table S1.pdf]

Table S1. Detection parameters of the biomarker analytes

Kit number	Kit 1		Kit 2										
**Detection (pg/ml)**	CRP[1]	Angiogenin	ST2	MMP-1	CHI3L1/ YKL-40	Angio-poietin-2	IL-1RA	CCL20/ MIP-3α	RAGE[4]	Periostin	VCAM1	CCL17/ TARC	TREM1
**upper level[2]**	27,810	5,510	74,810	12,090	97,850	17,660	6,790	1,990	37,650	298,260.0	1,890,780	27,830	11,640
**lower level[2]**	38.15	7.56	102.62	16.58	402.67	24.22	9.31	2.73	51.65	409.14	2,593.66	38.18	15.97
**Dilution**	1:2000	1:2000	1:2	1:2	1:2	1:2	1:2	1:2	1:2	1:2	1:2	1:2	1:2
**Kit Cat No[3]**	RnD cat. LXSAHM		RnD cat. LXSAHM										

Kit number	Kit 3											
**Detection (pg/ml)**	TNFRI/ TNFRSF1A	TNF RII/ TNFRSF1B	SP-D	MMP-3	MMP-7	MMP-8	MMP-12	MMP-13	IL-8/ leukocyte[5]	IL-6Ra	GP130	CCL11/ Eotaxin
**upper level[2]**	13,570	2,960	66,220	20,000	60,020	54,080	9,360	39,500	830	25,840	112,450	12,370
**lower level[2]**	18.61	4.06	90.84	27.43	82.33	74.18	12.84	54.18	1.14	35.45	154.25	16.97
**Dilution**	1:2	1:2	1:2	1:2	1:2	1:2	1:2	1:2	1:2	1:2	1:2	1:2
**Kit Cat No[3]**	RnD cat. LXSAHM											

Kit number	Kit 4		Kit 5				Kit 6		
**Detection (pg/ml)**	MMP-2	TIMP-1	TNFα	IL-33	IL-1β	IL-6	IL-13	IL-4	IL-8 Endothelial[6]
**upper level[2]**	68,640	8,990	5,000	11,300	3,800	2,700	1,000	7,500	1,250
**lower level[2]**	94.16	12.33	0.60	1.36	0.46	0.33	0.244	1.831	0.305
**Dilution**	1:50	1:50	1:2 or 1:3[6]	1:2 or 1:3	1:2 or 1:3	1:2 or 1:3	1:2 or 1:3[7]	1:2 or 1:3	1:2 or 1:3
**Kit Cat No[3]**	RnD cat. LXSAHM		RnD cat. FCSTM14				Millipore cat. HSTCMAG-28SK		

[1]CRP: C reactive protein; CCL: CC motif chemokine ligand; CHI3L1/YKL-40: chitinase 3 like 1; GP130: glycoprotein 130 (IL-6 receptor component); IL-6Ra (IL-6 receptor component); IL: Interleukin; IL-1RA: IL-1 receptor antagonist; IL-33: IL-1 family member; IL-33 receptor, ST2: Suppression Of Tumorigenicity2; MMP: matrix metallo-proteinase; RAGE: advanced glycosylation end product-specific receptor; SP-D: Surfactant Protein D; TIMP: tissue inhibitor of metallo-proteinases; TNF: tumor necrosis factor; TNFR: TNF receptor components; TREM1: Triggering Receptor Expressed On Myeloid Cells 1; VCAM: Vascular Cell Adhesion Molecule

[2]pg/ml

[3]Catalogue number of the kits, either purchased from RnD Systems, or Millipore.

[4]RAGE, detection of the endogenous soluble form and the proteolytically cleaved form of full-length RAGE

[5]IL-8, detection reagents raised specifically against the leukocyte form of IL-8 (RnD Systems)

[6]IL-8, detection reagents raised specifically against the endothelial form of IL-8 (Millipore)

[7]most tests were performed at 1:2 dilution, confirmative tests were performed at 1:3 dilution with similar (confirmatory) results
