# Supplementary material for: Molecular Clustering Analysis of Blood Biomarkers in World Trade Center Exposed Community Members with Persistent Lower Respiratory Symptoms
Source: Int J Environ Res Public Health. 2022 Jul 1;19(13):8102. doi: 10.3390/ijerph19138102 (PMC9266229; doi:10.3390/ijerph19138102)
Supplement: Supplementary file 1 [file ijerph-19-08102-s001.zip › Supplemental Table S2.pdf]

Supplemental Table S2. Clinical characteristics of WTCS cluster 1 and WTCS cluster 2.

	**Level**	**WTCS cluster 1**	**WTCS cluster 2**	**P**	**Test**	**Missing**
n		19	31			
Age, mean (SD)		53.7 (9.0)	55.9 (11.0)	0.456		0
Gender, n (%)	F	17 (89.5)	18 (58.1)	0.042		0
	M	2 (10.5)	13 (41.9)			
BMI, mean (SD)		30.3 (5.3)	29.8 (4.7)	0.75		0
Ethnicity, n (%)	Black	7 (36.8)	7 (22.6)	0.003	exact	0
	Latino	3 (15.8)	19 (61.3)			
	Other	3 (15.8)	0 (0.0)			
	White	6 (31.6)	5 (16.1)			
Education, n (%)	Grade school (up to 6th grade)	0 (0.0)	3 (9.7)	0.11		0
	High school (12th grade)	4 (21.1)	12 (38.7)			
	More than high school	15 (78.9)	16 (51.6)			
Currently smoking, n (%)	No	19 (100.0)	30 (96.8)	1	exact	0
	Yes	0 (0.0)	1 (3.2)			
PrePre BD FVC % pred[2] (mean (SD))		96.8 (15.7)	92.9 (14.7)	0.386		2
Post BD FVC % p		98.2 (13.9)	94.4 (15.4)	0.404		2
Pre BD FEV_1 % pred[3] (mean (SD))			91.6 (13.2)	0.512		2
Post BD FEV_1 % pred[3] (mean (SD))		96.7 (12.5)	94.2 (14.9)	0.554		2
IgE (IU/ml), mean (SD)		57.2 (48.9)	100.8 (187.2)	0.326		0
