# Supplementary material for: Molecular Clustering Analysis of Blood Biomarkers in World Trade Center Exposed Community Members with Persistent Lower Respiratory Symptoms
Source: Int J Environ Res Public Health. 2022 Jul 1;19(13):8102. doi: 10.3390/ijerph19138102 (PMC9266229; doi:10.3390/ijerph19138102)
Supplement: Supplementary file 1 [file ijerph-19-08102-s001.zip › Supplemental Table S3.pdf]

Supplemental Table S3. Comparison of Age, BMI, Gender and Race/Ethnicity between WTCS groups defined by WTC exposure categories

WTC Dust at 9/11	Level	No	Yes	P[1]
Age, mean (SD)		55.1 (10.5)	56.1 (10.0)	0.508
BMI, mean (SD)		29.9 (5.0)	30.7 (5.8)	0.466
Gender, n (%)	F	27 (75.0)	24 (64.9)	0.446
	M	9 (25.0)	13 (35.1)	
Race/Ethnicity, n (%)	Black	9 (25.0)	11 (29.7)	0.518
	Latino	17 (47.2)	16 (44.2)	
	Other	4 (11.1)	1 (2.7)	
	White	6 (16.7)	9 (24.3)	
**WTC Participant Category**	**Level**	**Resident**	**Worker**	**P**
Age, mean (SD)		53.3 (12.8)	56.2 (9.3)	0.484
BMI, mean (SD)		30.6 (6.9)	30.3 (5.0)	0.679
Gender, n (%)	F	11 (68.8)	40 (70.2)	1.000
	M	5 (31.3)	17 (29.8)	
Race/Ethnicity, n (%)	Black	3 (18.8)	17 (29.8)	0.088
	Latino	5 (31.3)	28 (49.1)	
	Other	3 (18.8)	2 (3.5)	
	White	5 (31.3)	10 (17.5)	
**Workplace with Ash**	**Level**	**No**	**Yes**	**P**
Age, mean (SD)		56.7 (9.4)	55.6 (8.8)	0.961
BMI, mean (SD)		30.2 (5.1)	30.0 (5.8)	0.865
Gender, n (%)	F	16 (76.2)	24 (64.9)	0.556
	M	5 (23.8)	13 (35.1)	
Race/Ethnicity, n (%)	Black	6 (28.6)	11 (29.7)	0.229
	Latino	7 (33.3)	20 (54.1)	
	Other	2 (9.5)	1 (2.7)	
	White	6 (28.6)	5 (13.5)	

[1] Age and BMI were compared using the independent, two-tailed Mann-Whitney test; gender and race/ethnicity were compared using the two-sided Fisher exact test.
