# Supplementary figures and images for: Molecular Clustering Analysis of Blood Biomarkers in World Trade Center Exposed Community Members with Persistent Lower Respiratory Symptoms
Source: Int J Environ Res Public Health. 2022 Jul 1;19(13):8102. doi: 10.3390/ijerph19138102 (PMC9266229; doi:10.3390/ijerph19138102)

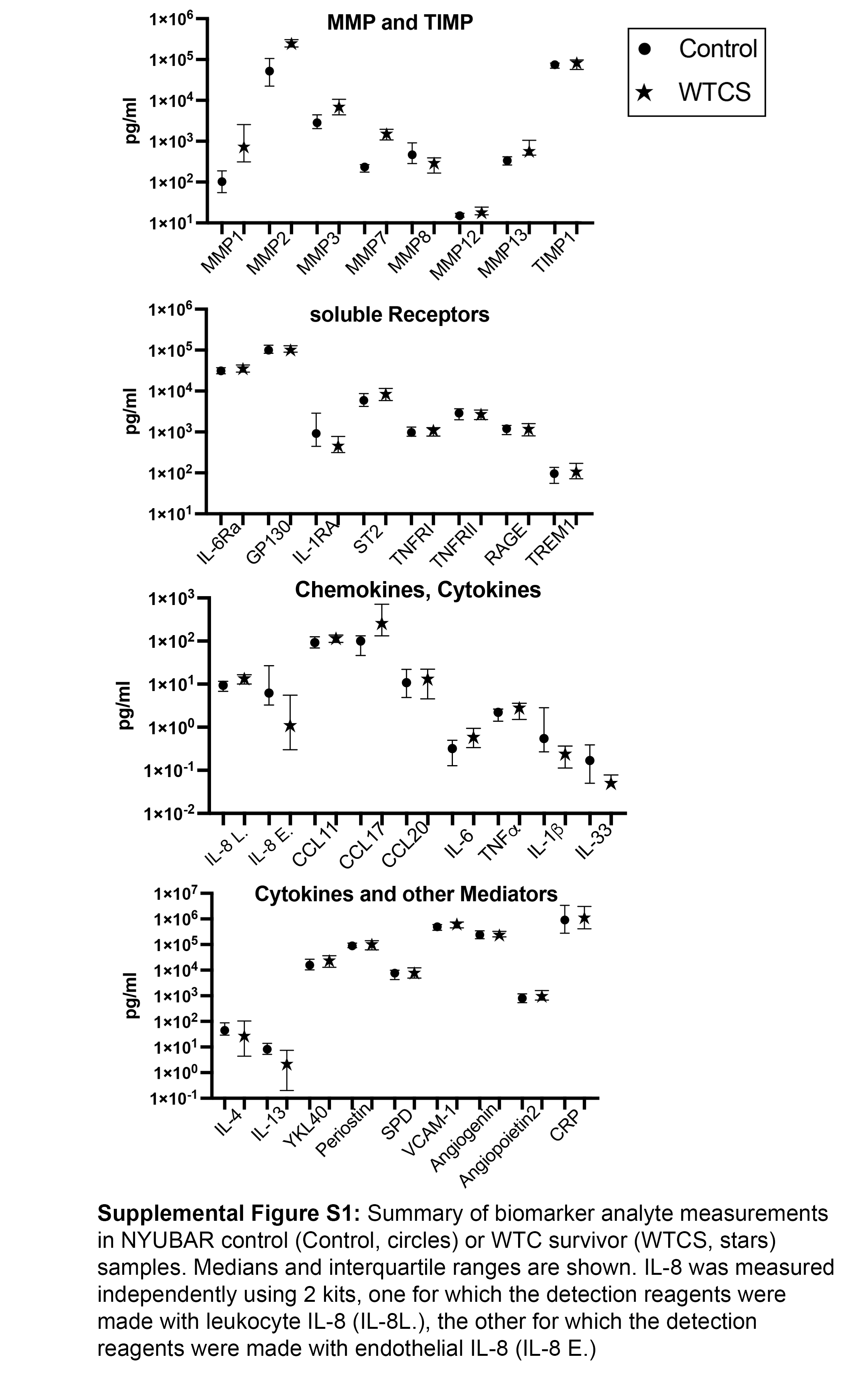

Supplement: Supplementary file 1 [file ijerph-19-08102-s001.zip › SupplementalFig S1.png]

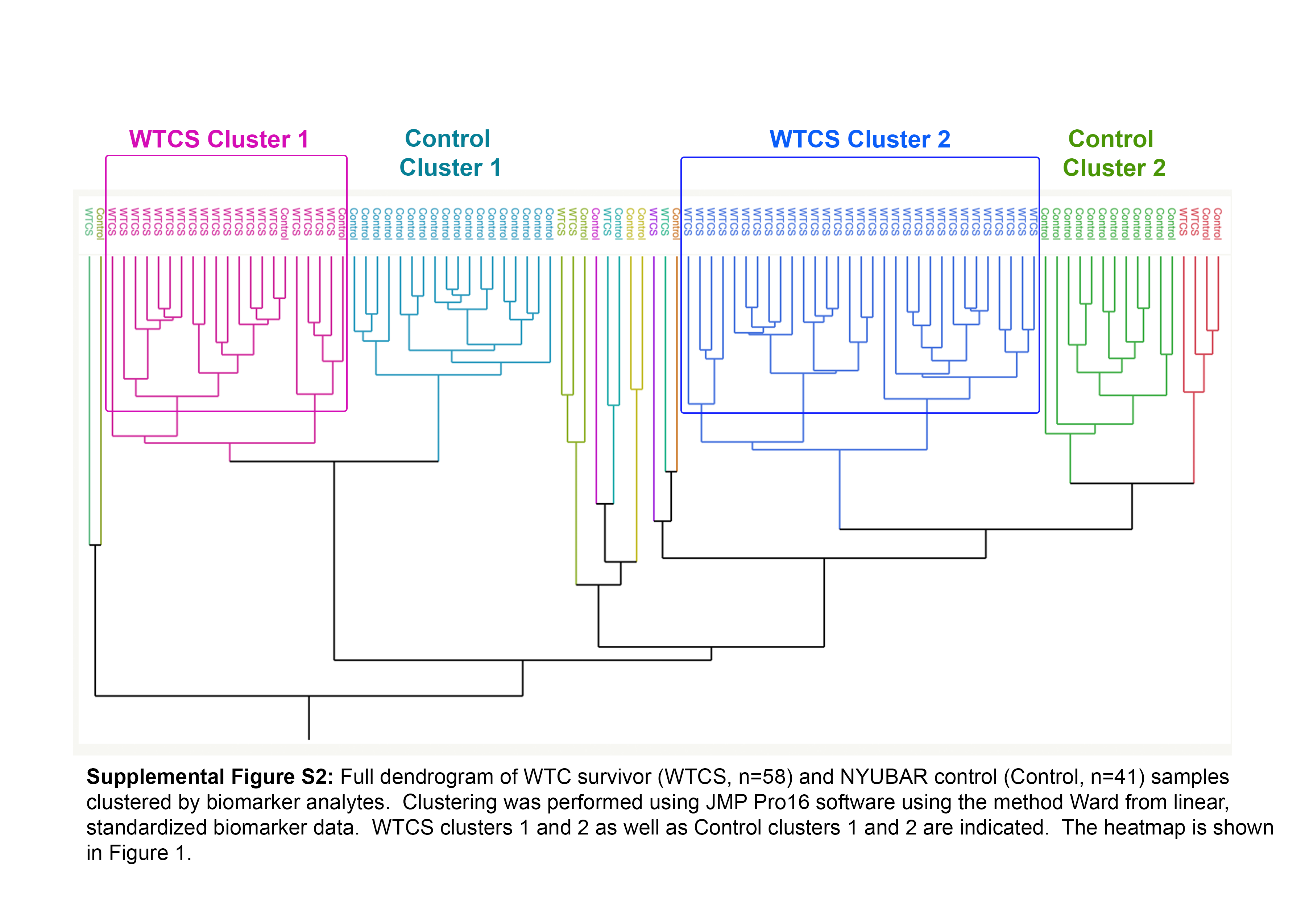

Supplement: Supplementary file 1 [file ijerph-19-08102-s001.zip › SupplementalFig S2.png]

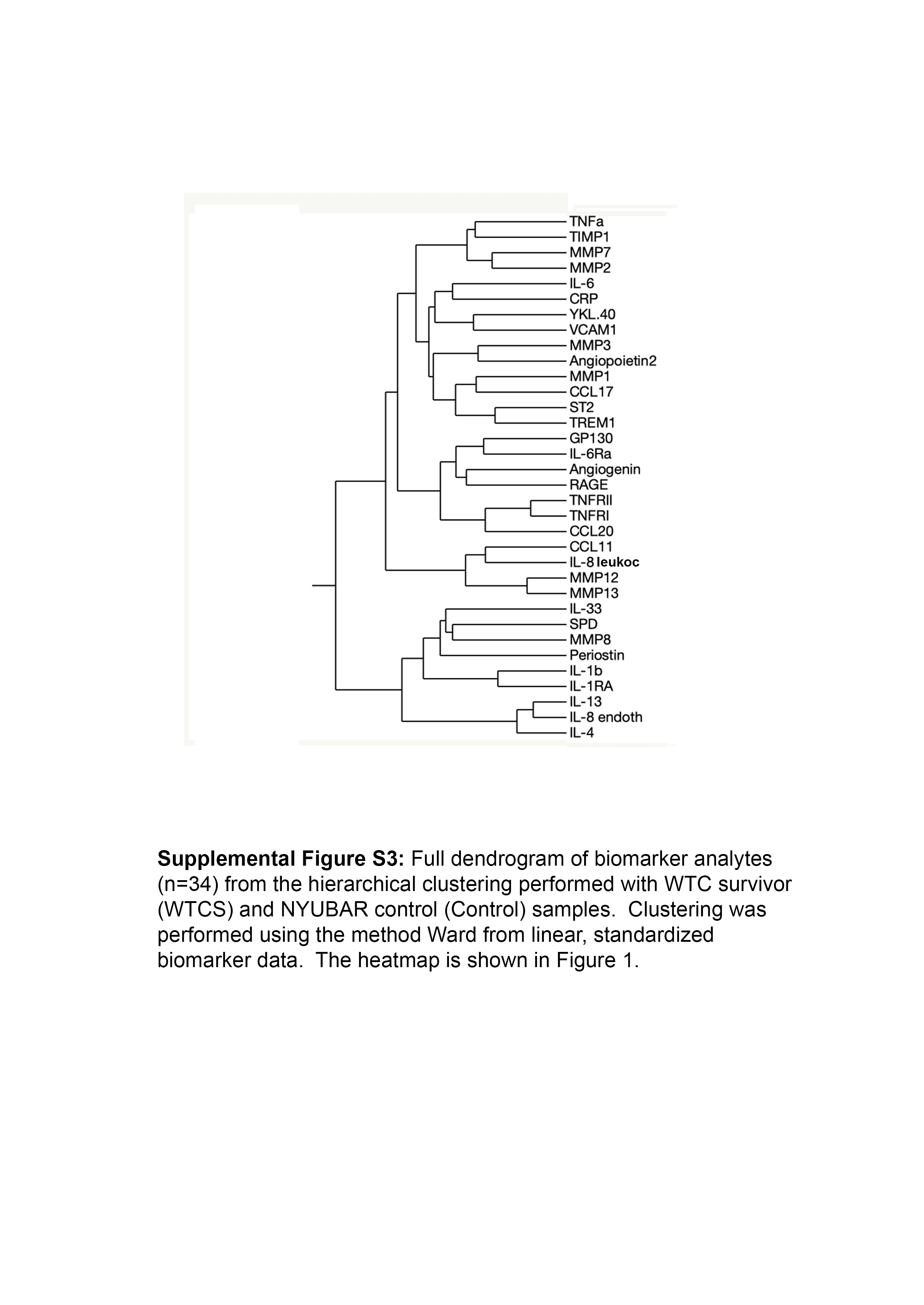

Supplement: Supplementary file 1 [file ijerph-19-08102-s001.zip › SupplementalFig S3.png]

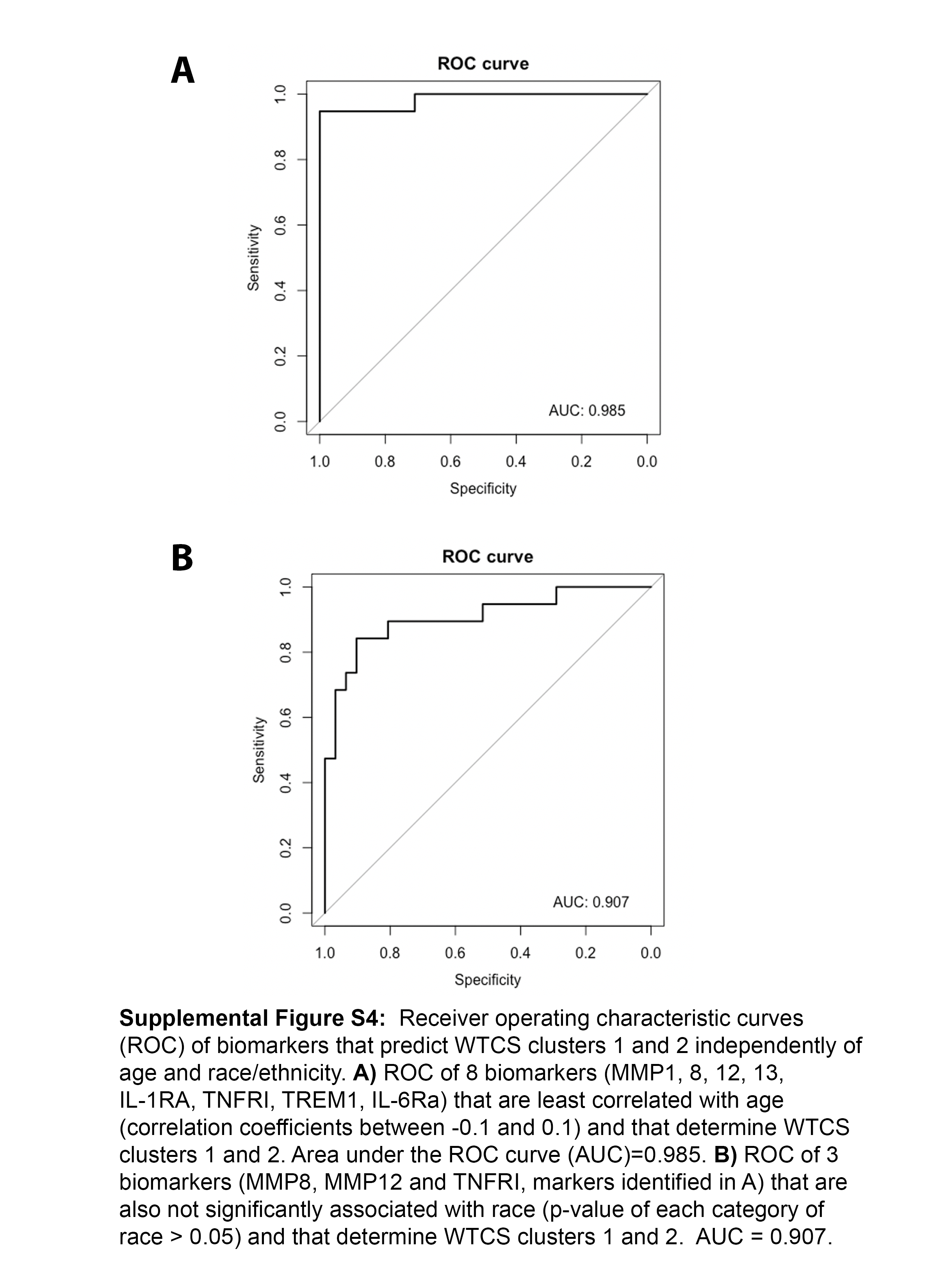

Supplement: Supplementary file 1 [file ijerph-19-08102-s001.zip › SupplementalFig S4.png]

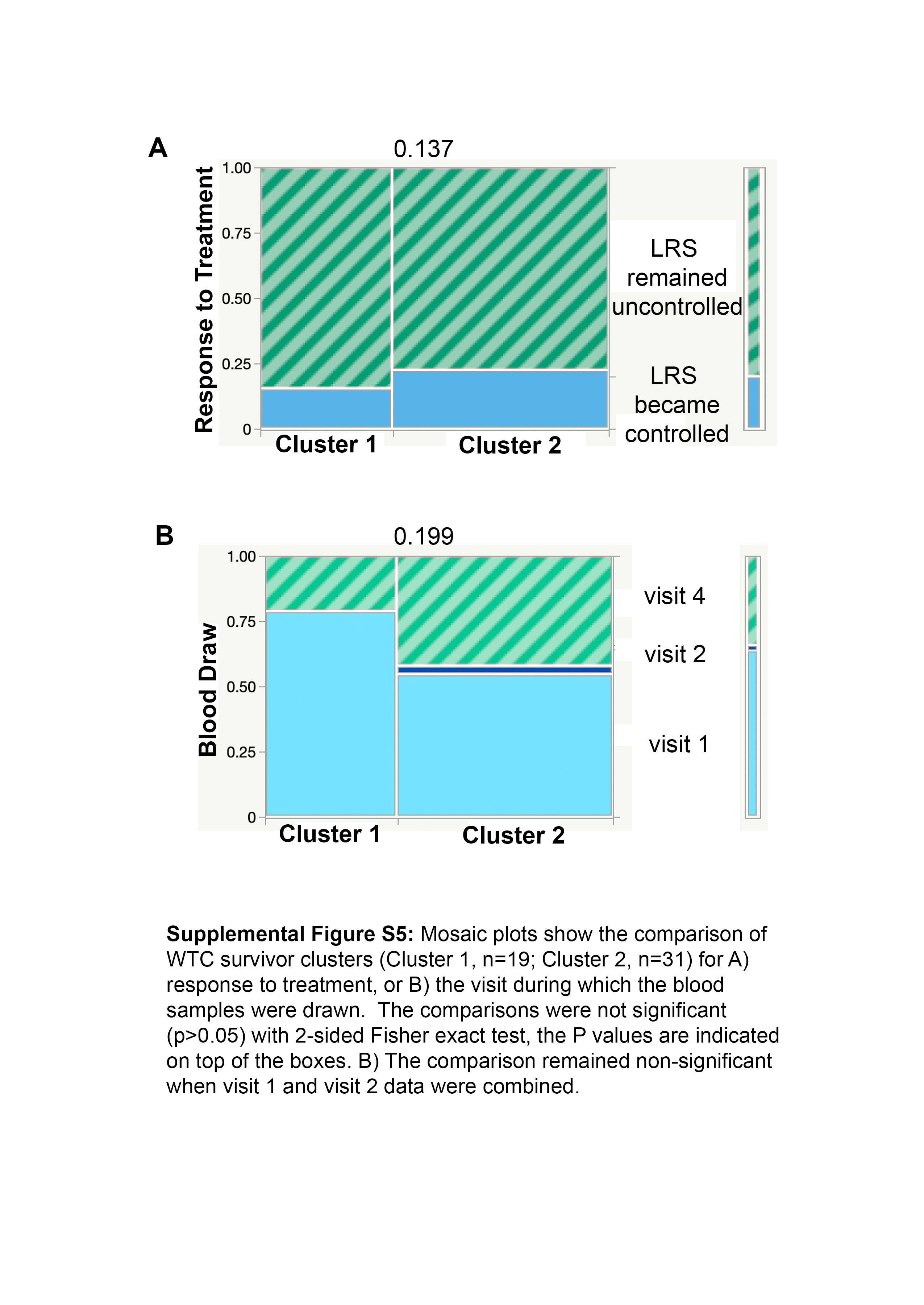

Supplement: Supplementary file 1 [file ijerph-19-08102-s001.zip › SupplementalFig S5.png]
